# Supplementary material for: Analysis of PPARγ Signaling Activity in Psoriasis
Source: Int J Mol Sci. 2021 Aug 10;22(16):8603. doi: 10.3390/ijms22168603 (PMC8395241; doi:10.3390/ijms22168603)
Supplement: Supplementary file 1 [file ijms-22-08603-s001.zip › Supplemental materials_Analysis of PPARg signaling activity in psoriasis/Pathway models/Models images and html files/PPARG inhibits phenotypes assotiated with psoriasis/142112.html]

cell proliferation


# Cell Process cell proliferation

|  |  |
| --- | --- |
| URN | urn:agi-gocellproc:0008283 |
| Total Entities | 224 |
| Connectivity | 28216 |
| Name | cell proliferation |
| ObjectType | Cell Process |

---

|  |  |
| --- | --- |
| ChildConcepts | trophoblast growth |
|  | parathyroid proliferation |
|  | epithelial cell proliferation |
|  | endometrium proliferation |
|  | epidermal proliferation |
|  | kidney mesangial cell proliferation |
|  | neuroproliferation |
|  | T-cell proliferation |
|  | lymphocyte proliferation |
|  | natural killer cell proliferation |
|  | endothelial cell proliferation |
|  | neuroblast proliferation |
|  | glia proliferation |
|  | septum cell proliferation |
|  | leukocyte proliferation |
|  | PBMC proliferation |
|  | myocyte proliferation |
|  | thymocyte proliferation |
|  | B-cell proliferation |
|  | keratinocyte proliferation |
|  | gamma-delta T-cell proliferation |
|  | alpha-beta T-cell proliferation |
|  | fibroblast proliferation |
|  | SMC proliferation |
|  | urothelial cell proliferation |
|  | myoblast proliferation |
|  | trophectoderm cell proliferation |
|  | natural killer T cell proliferation |
|  | osteoclast proliferation |
|  | mesenchymal cell proliferation |
|  | Schwann cell proliferation |
|  | satellite cell proliferation |
|  | osteoblast proliferation |
|  | chondrocyte proliferation |
|  | germ cell proliferation |
|  | pancreatic beta cell proliferation |
|  | neural precursor cell proliferation |
|  | adipocyte proliferation |
|  | oligodendrocyte progenitor proliferation |
|  | mast cell proliferation |
|  | hair follicle cell proliferation |
|  | hematopoietic stem cell proliferation |
|  | stem cell proliferation |
|  | glomerular mesangial cell proliferation |
|  | pancreatic stellate cell proliferation |
|  | hepatocyte proliferation |
|  | lens epithelial cell proliferation |
|  | Sertoli cell proliferation |
|  | cardiomyocyte proliferation |
|  | regulatory T-cell proliferation |
|  | lung fibroblast proliferation |
|  | megakaryocyte progenitor cell proliferation |
|  | umbilical vein endothelial cell proliferation |
|  | Tfh cell proliferation |
|  | myofibroblast proliferation |
|  | CD8+ T-cell proliferation |
|  | CD4+ T-cell proliferation |
|  | Th1 cell proliferation |
|  | immunocompetent cell proliferation |
|  | neutrophil proliferation |
|  | macrophage proliferation |
|  | hematopoietic cell proliferation |
|  | activated T-cell proliferation |
|  | monocyte proliferation |
|  | cancer stem cell proliferation |
|  | myeloid cell proliferation |
|  | kidney mesangial cell proliferation |
|  | cytotoxic T-cell proliferation |
|  | capillary endothelial cell proliferation |
|  | erythroid cell proliferation |
|  | B-lymphoid precursor cell proliferation |
|  | microglia proliferation |
|  | colony forming cell proliferation |
|  | Th17 cell proliferation |
|  | bone marrow macrophage proliferation |
|  | memory T-cell proliferation |
|  | neutrophil precursor proliferation |
|  | memory B-cell proliferation |
|  | dermal microvascular endothelial cell proliferation |
|  | spleen lymphocyte proliferation |
|  | immune system cell proliferation |
|  | megakaryocyte proliferation |
|  | dendritic cell proliferation |
|  | effector T-cell proliferation |
|  | activated B-cell proliferation |
|  | naive T-cell proliferation |
|  | eosinophil proliferation |
|  | erythroblast proliferation |
|  | mature B-cell proliferation |
|  | alveolar macrophage proliferation |
|  | activated CD8+ T-cell proliferation |
|  | CD8+ naive T-cell proliferation |
|  | germinal center B-cell proliferation |
|  | myeloid progenitor cell proliferation |
|  | CD8+ memory T-cell proliferation |
|  | bone marrow derived mesenchymal stem cell proliferation |
|  | naive B-cell proliferation |
|  | granulocyte precursor cell proliferation |
|  | helper T-cell proliferation |
|  | erythroid precursor cell proliferation |
|  | peripheral lymphocyte proliferation |
|  | blast cell proliferation |
|  | marginal zone B-cell proliferation |
|  | Th2 cell proliferation |
|  | plasma cell proliferation |
|  | cancer-associated fibroblast proliferation |
|  | decidual natural killer cell proliferation |
|  | histiocyte proliferation |
|  | Kuppfer cell proliferation |
|  | plasmablast proliferation |
|  | brain capillary endothelial cell proliferation |
|  | CD4+ memory T-cell proliferation |
|  | blood platelet proliferation |
|  | M2 macrophage proliferation |
|  | cytokine-induced killer cell proliferation |
|  | myeloid derived suppressor cell proliferation |
|  | regulatory B-cell proliferation |
|  | double negative thymocyte proliferation |
|  | bone marrow stem cell proliferation |
|  | T-lymphoblast proliferation |
|  | lymphoid progenitor cell proliferation |
|  | bone marrow derived mononuclear cell proliferation |
|  | invariant natural killer T-cell proliferation |
|  | fibrocyte proliferation |
|  | Langerhans cell proliferation |
|  | antigen specific cytotoxic T-cell proliferation |
|  | eosinophil precursor proliferation |
|  | sinusoidal cell proliferation |
|  | bone marrow derived mast cell proliferation |
|  | large granular lymphocyte proliferation |
|  | CD4+ naive T-cell proliferation |
|  | granulocyte proliferation |
|  | mast cell precursor proliferation |
|  | T-lymphoid precursor cell proliferation |
|  | lymphoblast proliferation |
|  | cytotoxic lymphocyte proliferation |
|  | tumor-infiltrating lymphocyte proliferation |
|  | bone marrow stromal cell proliferation |
|  | pluripotent bone marrow stem cell proliferation |
|  | granulocyte/macrophage progenitor proliferation |
|  | activated CD4+ T-cell proliferation |
|  | double positive thymocyte proliferation |
|  | plasmacytoid dendritic cell proliferation |
|  | conventional dendritic cell proliferation |
|  | mononuclear phagocyte proliferation |
|  | inducible CD4+CD25+ regulatory T-cell proliferation |
|  | promyelocyte proliferation |
|  | antigen-presenting cell proliferation |
|  | follicular B-cell proliferation |
|  | resting B-cell proliferation |
|  | peritoneal macrophage proliferation |
|  | B-1b B-cell proliferation |
|  | promonocyte proliferation |
|  | platelet precursor cell proliferation |
|  | B-1 B-cell proliferation |
|  | follicular dendritic cell proliferation |
|  | class switched memory B-cell proliferation |
|  | T2 B-cell proliferation |
|  | effector memory CD8+ T-cell proliferation |
|  | reticulocyte proliferation |
|  | CD4+ CD25+ regulatory T-cell proliferation |
|  | basophil proliferation |
|  | effector memory CD4+ T-cell proliferation |
|  | immature B-cell proliferation |
|  | Tr1 cell proliferation |
|  | foam cell proliferation |
|  | prolymphocyte proliferation |
|  | M1 macrophage proliferation |
|  | anergic T-cell proliferation |
|  | lymphokine-activated killer cell proliferation |
|  | pre-thymocyte proliferation |
|  | phagocyte proliferation |
|  | killer cell proliferation |
|  | B-1a B-cell proliferation |
|  | CD8+ regulatory T-cell proliferation |
|  | CD4+ cytotoxic T-cell proliferation |
|  | B-lymphoblast proliferation |
|  | CD30+ T-cell proliferation |
|  | megakaryoblast proliferation |
|  | natural helper cell proliferation |
|  | antibody-producing cell proliferation |
|  | Vg9/Vd2 T-cell proliferation |
|  | effector memory T-cell proliferation |
|  | myeloblast proliferation |
|  | multipotent bone marrow stem cell proliferation |
|  | senescent CD4+ T-cell proliferation |
|  | proerythroblast proliferation |
|  | intraglomerular mesangial cell proliferation |
|  | CD5+ T-cell proliferation |
|  | Th0 cell proliferation |
|  | null cell proliferation |
|  | mononuclear leukocyte proliferation |
|  | leukocyte precursor proliferation |
|  | myelocyte proliferation |
|  | CD28- CD8+ regulatory T-cell proliferation |
|  | central memory CD4+ T-cell proliferation |
|  | epithelioid cell proliferation |
|  | perivascular epithelioid cell proliferation |
|  | burst forming unit E proliferation |
|  | activated dendritic cell proliferation |
|  | natural killer cell precursor proliferation |
|  | tumor infiltrating macrophage proliferation |
|  | adipose macrophage proliferation |
|  | CD4+ thymocyte proliferation |
|  | peripheral blood stem cell proliferation |
|  | neutrophil band cell proliferation |
|  | senescent T-cell proliferation |
|  | peritoneum mast cell proliferation |
|  | Th9 cell proliferation |
|  | basophilic erythroblast proliferation |
|  | pleural macrophage proliferation |
|  | CD11c+ CD8+ T-cell proliferation |
|  | CD122+ CD8+ regulatory T-cell proliferation |
|  | centroblast proliferation |
|  | transitional B-cell proliferation |
|  | exhausted T-cell proliferation |
|  | activated natural killer cell proliferation |
|  | Th22 cell proliferation |
|  | nuocyte proliferation |
|  | central memory CD8+ T-cell proliferation |
|  | CD103+ CD8+ T-cell proliferation |
|  | monocytoid B-cell proliferation |
|  | bone marrow derived dendritic cell proliferation |
|  | CFU-GEMM proliferation |

---

|  |  |
| --- | --- |
| Pathway | Alzheimer's Disease |
|  | Osteoarthritis |
|  | Thyroid Hormones Common non-Genomic Effects in Hyperthyroidism |
|  | Adrenoceptors Physiological Effects |
|  | PRL and CSH Action in Mammary Gland |
|  | Insulin Signaling |
|  | Prolactin Production |
|  | LH and FSH Signaling and Steroidogenesis |
|  | CD8+ T-Cell Activation |
|  | T-Cell Receptor Signaling |
|  | Lipoxin A4/FPR2-Related Neutrophil Depression |
|  | AHR in Intestinal Cell Antimicrobial Barrier Maintenance |
|  | Eicosanoids in Myeloid Cell Activation |
|  | Neutrophil Activation via LTB4/Polyisoprenyl Phosphate Signaling |
|  | Mast-Cell Activation without Degranulation through CYSLTR1/CYSLTR2 Signaling |
|  | Vascular Smooth Muscle Cell/Pericyte Differentiation and Proliferation |
|  | Tight Junction Assembly (Occludin) |
|  | Tight Junction Assembly (Claudins) |
|  | Adherens Junction Assembly (Cadherins) |
|  | SCF/FBXW7 Complex |
|  | Endothelin Signaling in Arterial Hypertension |
|  | Endothelial Cell Dysfunction in Arterial Hypertension |
|  | Aldosterone Synthesis Chronic Regulation |
|  | BMP/TGF-beta Signaling Impairment in Pulmonary Hypertension |
|  | Endothelial Cell Dysfunction in Pulmonary Hypertension |
|  | Smooth Muscle Cell Dysfunction in Pulmonary Hypertension |
|  | BMP2 and Canonical WNT Signaling in Pulmonary Artery Endothelial Cells |
|  | Renin-Angiotensin-Aldosterone System in Myocardial ischemia |
|  | Sebocyte Proliferation in Acne Vulgaris |
|  | Androgen Induced Sebocyte Hyperfunction |
|  | Keratinocyte Proliferation in Acne Vulgaris |
|  | Telogen Maintenance in Androgenic Alopecia |
|  | Androgens in Sebocyte Maturation |
|  | Androgens Promote Scalp Dermal Papilla Regression |
|  | Androgens Synthesis in Sebocytes |
|  | Atopic Dermatitis Onset |
|  | WNT Signaling in Melanoma |
|  | IL17 Signaling in Psoriasis |
|  | IL22 Induces Keratinocyte Proliferation in Psoriasis |
|  | T-Cell Cytotoxic Response against Melanocytes in Vitiligo |
|  | Donohue Syndrome Progression (Hypothesis) |
|  | Tubular Cell Dysfunction in Progressive Diabetic Nephropathy |
|  | Endothelial Cell Dysfunction in Progressive Diabetic Nephropathy |
|  | Endothelial Cell Dysfunction in Early Diabetic Nephropathy |
|  | Mesangial Cell Dysfunction in Diabetic Nephropathy |
|  | Microangiopathy in Diabetic Neuropathy |
|  | Proliferative Diabetic Retinopathy |
|  | Hepatic Stellate Cells in non-Alcoholic Fatty Liver Disease |
|  | beta-Cell Mass Regulation |
|  | beta-Cell Function Inhibition by Ciclosporine and Tacrolimus (Rodent Model) |
|  | FOXO1 and SREBP-1C Role in beta-Cell Suppression (Rodent Model) |
|  | CDC73 Mutations Cause Hyperparathyroidism |
|  | MEN1 Mutation in Hyperparathyroidism |
|  | RET1 Mutation in Hyperparathyroidism |
|  | Ethanol Induced Efects in Hyperprolactinemia |
|  | Thyroid Dysfunction |
|  | GLIS3 Targets in Thyroid Dysgenesis (Hypothesis) |
|  | Thyroid Stimulating Hormone (TSH) Resistance in Congenital Hypothyroidism |
|  | Overt Hypothyroidism, Primary |
|  | CD4+T-Cell Response in Celiac Disease |
|  | Cetuximab Resistance in Colorectal Cancer |
|  | Metastatic Colorectal Cancer |
|  | GUCY2C Signaling in Colorectal Cancer |
|  | EGFR Nuclear Signaling in Colorectal Cancer |
|  | CagA Phosphorylation Dependent Signaling |
|  | Neurogenic Transcription Factors Role in Hirschsprung Disease |
|  | EDN, NRG, NRTN, and GDNF/RET Signaling in Hirschsprung Disease |
|  | Peutz-Jeghers Syndrome |
|  | RARA Signaling in Acute Myeloid Leukemia (M3) |
|  | Acute Myeloid Leukemia |
|  | Myeloid Cell Differentiation Block in Leukemia |
|  | B-Cell Acute Lymphoblastic Leukemia |
|  | B-Cell Chronic Lymphocytic Leukemia |
|  | Chronic Myeloid Leukemia |
|  | Diffuse Large-B-Cell Lymphoma |
|  | Diffuse Large-B-Cell Lymphoma ABC Subtype |
|  | Lymphoma, GCB Subtype |
|  | Primary Mediastinal B-cell Lymphoma (PMBL) Subtype |
|  | Follicular Lymphoma |
|  | Hodgkin Lymphoma |
|  | Mantle Cell Lymphoma |
|  | mTOR/NF-kB/BCR Signaling Disregulation in Mantle Cell Lymphoma |
|  | WNT Canonical Signaling in Mantle Cell Lymphoma |
|  | Hedgehog Signaling in Mantle Cell Lymphoma |
|  | Mucosa-Associated Lymphoid Tissue (MALT) Lymphoma |
|  | Splenic Marginal Zone Lymphoma |
|  | TNFR/NF-kB Classical Pathway in Multiple Myeloma Plasma Cells |
|  | IL6/IGF1/VEGFA Signaling in Multiple Myeloma |
|  | Multiple Myeloma |
|  | NOTCH Signaling in Multiple Myeloma |
|  | TNFR/NF-kB Alternative Pathway in Multiple Myeloma Plasma Cells |
|  | T-Cell Acute Lymphoblastic Leukemia |
|  | T-Cell Genetic Rearrangements in Acute Lymphoblastic Leukemia |
|  | NOTCH Signaling in Hepatocellular Carcinoma |
|  | WNT Signaling in Hepatocellular Carcinoma |
|  | Hepatocellular Carcinoma |
|  | TP53 Signaling in Hepatocellular Carcinoma |
|  | Growth Factor Signaling in Hepatocellular Carcinoma |
|  | Pancreatic Ductal Carcinoma |
|  | Growth Factor Signaling in Pancreatic Neoplasms |
|  | TP53 Signaling in Pancreatic Neoplasms |
|  | Pancreatic Neuroendocrine Tumors |
|  | STK11 Signaling in Pancreatic Neoplasms |
|  | TRPM7/8 in Pancreatic Adenocarcinoma |
|  | TGFBR Signaling in Pancreatic Neoplasms |
|  | Osteoclast Activation in Gout |
|  | Ovulation Block |
|  | Polycystic Ovary Syndrome |
|  | FSH Action in Polycystic Ovary Syndrome |
|  | PTPN22 Role in Rheumatoid Arthritis |
|  | Synovial Fibroblast Proliferation in Rheumatoid Arthritis |
|  | Osteoclast Activation in Rheumatoid Arthritis |
|  | CCR5 Signaling in Macrophages |
|  | Diabetes Induced Periodontitis |
|  | Estrogen Effects on Pregnancy Gingivitis |
|  | Li-Fraumeni Syndrome |
|  | Hereditary Breast and Ovarian Cancer Syndrome |
|  | Cowden Syndrome |
|  | Dystrophin Glycoprotein Complex Signaling in Muscular Dystrophies |
|  | IGF1 Role in Muscle Hypertrophy |
|  | Myostatin-IGF1 Crosstalk in Skeletal Muscles in Muscular Dystrophies |
|  | Osteoblast Function Decline in Osteoporosis |
|  | Glioblastoma, Primary |
|  | Glioblastoma, Secondary |
|  | Astrocytoma |
|  | Glioma Stem Cell Program Activation |
|  | Ependymoma |
|  | Medulloblastoma |
|  | PHOX2B Effects in Neuroblastoma |
|  | Growth Factor Signaling in Neuroblastoma |
|  | Neuroblastoma |
|  | ALK Associated Neuroblastoma |
|  | Sonic Hedgehog Signaling in Neuroblastoma |
|  | non-Hereditary Genetic Rearrangements in Neuroblastoma |
|  | WNT Signaling in Breast Cancer |
|  | ERBB/VEGFR/Akt Signaling in Breast Cancer |
|  | IGF1R/AKT Signaling in Breast Cancer |
|  | ESR1/ERBB Positive Luminal Breast Cancer |
|  | Breast Cancer |
|  | ESR1 Signaling in Breast Cancer |
|  | NOTCH1 Signaling in Breast Cancer |
|  | Endometrial Cancer |
|  | Endometrioid Endometrial Cancer |
|  | Clear Cell Endometrial Cancer and Papillary Serous Endometrial Cancer |
|  | Coagulation Factors and PAR1/2 Receptors (F2R/F2RL1) in Endometriosis |
|  | Leptin Role in Endometriosis |
|  | Dioxin Role in Endometriosis |
|  | Endometrioid Ovarian Carcinoma |
|  | Clear Cell Ovarian Carcinoma |
|  | EGFR and PTAFR Signaling in Ovarian Cancer |
|  | High-Grade Serous Ovarian Carcinoma |
|  | Low-Grade Serous Ovarian Carcinoma |
|  | Ovarian Cancer |
|  | Mucinous Ovarian Carcinoma |
|  | INHA Gene Mutations in Granulosa Cell Proliferation |
|  | FOXL2 Mutation in Ovarian Dysgenesis |
|  | LH and FSH Signaling Impairment in Primary Ovarian Insufficiency |
|  | MIF Role in Cholesteatoma |
|  | EGF/cyclinD1 Signaling in Acquired Middle Ear Cholesteatoma |
|  | IL6/JAK/STAT3 Signaling in Cholesteatoma |
|  | HMGB1/RAGE Signaling in Middle Ear Cholesteatoma |
|  | Subepithelial Fibroblasts in Middle Ear Cholesteatoma |
|  | Cholesteatoma |
|  | HPV E6 and E7 Induced Block of Apoptosis in Keratinocytes |
|  | HPV Infection and Cancer |
|  | HPV E5 Regulates EDNRA and EGFR Signaling Pathway in Keratinocytes |
|  | Androgen Receptor/Akt Signaling |
|  | Fatty Acid Synthase (FASN) Signaling |
|  | Androgen Receptor/FKBP5 Signaling |
|  | Androgen Receptor/Prostate Specific Antigen Signaling in Prostate Cancer |
|  | Androgen Receptor/NKX3-1 Signaling |
|  | Kruppel-like Factor 6 Signaling in Prostate Cancer |
|  | Androgen Receptor/Beta-2-Microglobulin Signaling in Prostate Cancer |
|  | Prostate Cancer |
|  | beta-Catenin/Androgen Receptor Signaling in Prostate Cancer |
|  | RNase L Signaling in Prostate Cancer |
|  | Androgen Receptor/c-Met Signaling in Prostate Cancer |
|  | FOXM1 Signaling in Prostate Cancer |
|  | TRPM8 Effects in Prostate Cancer (Hypothesis) |
|  | Androgen Receptor/GJB1 Signaling |
|  | Steroids Induced Cataract |
|  | VEGFA in Neovascular Glaucoma |
|  | CAV1 Dependent Senescence of Lung Fibroblasts |
|  | WNT Signaling in Cystic Kidney Disease |
|  | Polycystins Mutation Effects in Cystic Kidney Disease |
|  | Endothelial Cell Dysfunction in Glomerulonephritis |
|  | Mesangial Cell Dysfunction in Glomerulonephritis |
|  | Interstitial Fibroblasts in Pyelonephritis |
|  | Endothelial Cell Dysfunction in Pyelonephritis |
|  | CD38 -> cADPR/Calcium Signaling |
|  | CD157 -> cADPR/Calcium Signaling |
|  | AGTR1 -> ARRB1/ARRB2 Signaling |
|  | Hedgehog Family -> ARRB1/ARRB2 Canonical Signaling |
|  | Tamoxifen Induced Endometrial Cancer |
|  | Clozapine Induced Granulocytopenia |
|  | EGFR Transactivation in Cancer and Non-Cancer Cells |
|  | TAM Receptor Signaling Activation in Cancer |
|  | PI3K/AKT/MTOR Signaling Activation in Cancer |
|  | DREAM Complex and FOXM1/MYBL2 Promote Cell Cycle Progression in Cancer |
|  | Hedgehog Signaling Activation in Cancer |
|  | Receptors and Adaptor Proteins Activated in Cancer |
|  | NOTCH Signaling Deregulation in Cancer |
|  | RAS/RAF/MAPK Signaling Activation in Cancer |
|  | WNT Canonical Signaling Activation in Cancer |
|  | NFKB Non-Canonical Signaling Activation in Cancer |
|  | STAT3 and NFkB Activate Inflammation-Induced Tumorigenesis |
|  | RAS/RAF/MAPK Signaling Activation by Blocking of Tumor Suppressors |
|  | Hedgehog Signaling Activation by Blocking of Tumor Suppressors |
|  | WNT Signaling Activation by Blocking of Tumor Suppressors |
|  | PI3K/AKT/MTOR Signaling Activation by Blocking of Tumor Suppressors |
|  | Hippo/YAP1 Signaling Deregulation in Cancer |
|  | NFKB Signaling Activation by Blocking of Tumor Suppressors |
|  | TERT/WNT Activation in Cancer |
|  | Oxidized LDL/OLR1 Activates Cell Proliferation in Cancer |
|  | ANGPT/TEK Stimulates Endothelial Cell Migration in Cancer |
|  | BSG (CD147) in Cancer Cells Motility, Invasion and Survival |
|  | Hyaluronic Acid, CD44 and HMMR in Cancer Cell Invasion and Survival |
|  | CDH2 Activation Promotes Cancer Cell Migration and Survival |
|  | Lipogenesis/Lipolysis Activation in Cancer Cells and Cancer-Associated Adipocytes |
|  | EZH2 Methyltransferase Role in Cancer |
|  | DNMT and MBD Families Activation in DNA Methylation in Cancer |
|  | Estrogens/ESR1 Genomic Canonical Signaling |
|  | Androgen Receptor non-Genomic Signaling |
|  | FOXO1 Signaling |
|  | WNT Canonical Signaling |
|  | Estrogens/ESR1 non-Genomic Signaling |
|  | EGFR Signaling |
|  | WNT Planar Cell Polarity (PCP) non-Canonical Signaling |
|  | Hedgehog Family Signaling |
|  | ROS in Angiotensin Mediated Cardiovascular Remodeling and Hypertrophy |
|  | n |
|  | cypa |
|  | PPARG inhibits phenotypes assotiated with psoriasis |
|  | H2A-K120, H2A-K119 and H3-K4 |
|  | H2A-K119 and H3-K27 |
|  | Epigenetic Modifications of H2A-K120, H2A-K119 and H3-K4 |
|  | Overview. Diseases of the genitourinary system (chapter 13) |
|  | Overview. Infectious and parasitic diseases (chapter 2) |
|  | 2\_Hyperseborrhea in acne vulgaris |
|  | 2\_Osteoblast-mediated bone demineralization |
|  | 1\_Formation of cysts in polycystic kidney disease |
|  | 3\_The role of interstitial fibroblasts in pyelonephritis |
|  | 3\_Mesangial cell dysfunction in glomerulonephritis |
|  | 2\_Progesterone resistance stimulates endometriotic cell survival |
|  | 1\_Local estrogen production stimulates ectopic endometrium proliferation |
|  | 3\_Enhanced angiogenesis in endometriosis |
|  | 2\_Impaired steroidogenesis in PCOS |
|  | 1\_Endothelial cells stimulate vasoconstriction and thrombosis in pulmonary hypertension |
|  | 3\_1\_Mucus accumulation in asthma |
|  | 1\_CD4+ T cell and epithelial cell response in celiac disease |
|  | 4\_Human papilloma virus E6 and E7 proteins promote epithelial cell survival |
|  | 2\_HIV - induced depletion of CD4+ T-cells |
|  | 3\_1\_Beta-cell proliferation and compensation: proliferation |
|  | 3\_3\_Beta-cell proliferation and compensation: recovery |
|  | 4\_1\_Insulin resistance: insulin signaling |
|  | 1\_Microangiopathy in diabetic neuropathy |
|  | 2\_1\_General pathways of thyroid hormone signaling |
|  | New Pathway |
|  | DIPG overview\_2 |

---

|  |  |
| --- | --- |
| MedScan ID | 4108283 |

---

|  |  |
| --- | --- |
| Alias | cell proliferations |
|  | proliferative cells |
|  | cells of the proliferative |
|  | cellular proliferations |
|  | cellular proliferation |
|  | hyperproliferations |
|  | cell proliferation |
|  | hyperproliferation |
|  | proliferating cell |
|  | proliferative responses |
|  | cells in proliferating |
|  | cell proliferation kinetics |
|  | kinetics of cell proliferation |
|  | proliferative response |
|  | proliferation of the cell |
|  | proliferations of the cell |
|  | proliferating cells |
|  | proliferation |
|  | proliferations |
|  | proliferative cell |
|  | hyper-proliferation |

---

|  |  |
| --- | --- |
| GO ID | 0008283 |

---
